# Outcomes of single institutional experience with Single Cross-Clamp technique

**DOI:** 10.1186/1749-8090-10-S1-A174

**Published:** 2015-12-16

**Authors:** Rajani Rajnish, David Rose, John Massey, Royan Richards, Mohamad N Bittar, Ionnis Dimarakis

**Affiliations:** 1 Department of Cardiothoracic Surgery Blackpool Victoria Hospital Whinney Heys Road, Blackpool, Lancashire, FY3 8NR, UK

## Background/Introduction

Minimizing cardiac surgery related mortality and furthermore morbidity remains at the center of patient focused care and quality improvement. We present our experience with single cross-clamp technique following coronary artery bypass graft surgery (CABG) with or without aortic valve replacement (AVR).

## Aims/Objectives

We hypothesize that a single cross clamp technique can improve survival whilst reducing associated morbidity.

## Method

All clinical data were prospectively collected with the study design and analysis being retrospective in nature. A single surgeon's procedures were included for the period October 2007 to January 2014

## Results

712 (male 77.5%) patients were operated on, with 609 (85.54%) being isolated CABG and 103 (14.46%) combined CABG with AVR. Average age in isolated CABG and combined groups were 66 and 73 years respectively. Average additive EuroScores were 3.5 and 7.29. 21.6% cases were performed by trainee or staff grade surgeon.

In hospital mortality was 0.16% in isolated CABG and 0% in CABG with AVR group. Postoperative complications in isolated CABG group included reoperation for bleeding or tamponade (2.13%), renal impairment requiring temporary haemodialysis (0.82%), and respiratory failure requiring reintubation (0.65%). We did not experience any cerebrovascular events. In the combined CABG with AVR group we experienced reoperation for bleeding or tamponade (2.91%), respiratory failure requiring reintubation (1.94%), and cerebrovascular events (1.94%).

Kaplan Meier survival at 1, 3, and 5 years was 98.35%, 95.85% and 92.61% (isolated CABG), and 96%, 86.4% and 79.4% (combined CABG with AVR).

## Discussion/Conclusion

Our preliminary study has demonstrated outcomes comparable to our nationally reported outcomes for the same time period. Single cross clamp technique for CABG with or without AVR is a safe and reproducible technique.

